# Digital Variance Angiography in Lower-Limb Angiography with Metal Implants

**DOI:** 10.1007/s00270-020-02697-x

**Published:** 2020-11-03

**Authors:** M. B. Bastian, A. M. König, S. Viniol, M. Gyánó, D. Szöllősi, I. Góg, J. P. Kiss, S. Osvath, K. Szigeti, A. H. Mahnken, R. P. Thomas

**Affiliations:** 1 Department of Diagnostic and Interventional Radiology, University Hospital Marburg, Philipps University of Marburg, Baldingerstrasse 1, 35043 Marburg, Germany; 2 Department of Interventional Radiology, Heart and Vascular Center, Semmelweis University, Budapest, Hungary; 3 Department of Biophysics and Radiation Biology, Semmelweis University, Budapest, Hungary; 4 Department of Vascular Surgery, Hungarian Defense Forces Medical Centre, Budapest, Hungary; 5 Kinepict Health Ltd, Budapest, Hungary

**Keywords:** Digital variance angiography, Contrast-to-noise ratio, Automated exposure control, Metal implants

## Abstract

**Purpose:**

The presence of metal implants may reduce angiographic image quality due to automated beam adjustments. Digital variance angiography (DVA) is reported to be superior to digital subtraction angiography (DSA) with increased contrast-to-noise ratio (CNR) and better image quality. The aim of the study was to evaluate whether DVA could counterbalance the image quality impairment of lower-limb angiographies with metal implants.

**Materials and Methods:**

From November 2019 to January 2020, 85 raw lower-limb iodine contrast angiograms of 12 patients with metal implants were processed retrospectively with DVA analyses. For objective comparison, CNR of DSA and DVA images was calculated and the ratio CNR_DVA_/CNR_DSA_ was determined. Visual image quality was evaluated in a paired comparison and by a five-grade Likert scale by three experienced radiologists.

**Results:**

The CNR was calculated and compared in 1252 regions of interest in 37 image pairs containing metal implants. The median ratio of CNR_DVA_/CNR_DSA_ was 1.84 with an interquartile range of 1.35–2.32. Paired comparison resulted in 84.5% in favour of DVA with an interrater agreement of 83.2% (Fleiss κ 0.454, *p* < 0.001). The overall image quality scores for DSA and DVA were 3.64 ± 0.08 and 4.43 ± 0.06, respectively (*p* < 0.001, Wilcoxon signed-rank test) with consistently higher individual ratings for DVA.

**Conclusion:**

Our small-sample pilot study shows that DVA provides significantly improved image quality in lower-limb angiography with metal implants, compared to DSA imaging. The improved CNR suggest that this approach could reduce radiation exposure for lower-limb angiography with metal implants.

**Level of Evidence:**

Level 4, case studies

## Introduction

Over the last decades, advances in endovascular technology and interventional therapy have broadened the percutaneous treatment options of peripheral vascular diseases offering an alternative to open surgery in patients with cardiovascular comorbidities. Balloon angioplasty and stenting are the mainstays of endovascular therapy in peripheral arterial disease (PAD), while drug-coated balloons and stents, adjunctive devices for crossing chronic total occlusions and debulking plaque with atherectomy, offer other treatment options. Adequate imaging of arteries is important in all these procedures. Traditionally vessels are made visible with injection of iodine contrast agents under X-ray radiation in digital subtraction angiography (DSA) technique [1, 2]. In DSA, a native image mask is recorded followed by a series of contrast images. The native mask is then subtracted from contrast image series to visualize only the structures filled with contrast agents, whilst leaving out other anatomical structures [3].

Iodinated contrast media (ICM) are most frequently used for angiographic guidance. Carbon dioxide (CO_2_) being a negative contrast is an alternative, when contraindications for ICM such as allergic reactions, renal problems and thyrotoxicosis exist [4–6]. An important goal of present-day research is the reduction in ICM usage and X-ray dose, to minimize ICM complications as well as the radiation exposure for patients and staff involved [7–10]. Lowering the radiation or ICM dose normally results in reduced image quality. Noise reduction algorithms are reported to provide a possible solution in this regard [8, 11].

Digital variance angiography (DVA), a recently developed technology based on the principle of kinetic imaging, is reported to gain more information from images created by penetrating radiation [12]. Improved image quality might be effectively used for dose management (radiation and ICM reduction) without compromising the diagnostic value of images.

The presence of metal implants in angiographic fields of interest could reduce image quality drastically. The implants reflect X-rays, resulting in more scattered radiation as well as noise around the implant, leading to automatic up-regulation of the applied radiation dose by automated beam adjustments [13]. This overexposure of X-rays could reduce the visualization of adjacent blood vessels drastically during angiographic procedures, and increased scattered radiation increases radiation exposure of the staff and patients. Reducing the collimation in the field of interest could reduce the overexposure to some extent. With reported noise reduction and improved image quality of DVA technology, our aim was to evaluate whether DVA could counterbalance the image quality impairment of lower-limb angiographies with metal implants.

## Materials and Methods

### Patient Selection

Twelve patients (mean age 82.3 (range 76–88) years) with metal implants undergoing lower-limb angiographic interventions for PAD from November 2019 to January 2020 were included in this retrospective observational study. The patients enrolled consisted of 5 men (mean age 82.3 (range 76–88) years) and 7 women (mean age 80.5 (range 79–84) years), and angiographic indications ranged from PADs Fountain Stages II–IV. The metal implants consisted of 7 hip total endoprosthesis, 3 knee total endoprosthesis, 1 gamma nail and 1 large knee arthrodesis.

### Study Design

Four pre- and four post-intervention image pairs were acquired for each patient**.** Minimum requirement was set as at least one image series with the presence of implant per patient. Out of total 96 image pairs, some images were excluded because of technical problems or inferior image quality of both image types. The study compared DVA and DSA images by the evaluation of CNRs and comparison of visual quality of both image groups retrospectively. The CNR was calculated and compared in 37 image pairs containing metal implants. The visual quality was compared in 85 image pairs, where 40 contained metal implants and 45 no implants. All 170 individual images were also evaluated by three radiologists using a five-grade Likert scale.

### Image Acquisition

The vascular access was via the femoral artery for all angiographic procedures, which were performed by a single interventional radiologist with more than 13 years of experience in interventional procedures. The intra-arterial contrast was injected manually in all cases. Depending on the location of the pathology, antegrade- or retrograde crossover approaches were decided by the angiographer. The diagnostic angiograms were performed with a 5F sheath/catheter, and interventions were performed with a 6F sheath. The angiographer judged contrast (Ultravist 300, Bayer Vital GmbH) volumes individually, where a dilution of 3:2 was commonly used. All procedures were performed in the angiography suite of the hospital (30 × 40 cm detector, Siemens Artis Zee, Siemens Healthineers AG, Erlangen, Germany) with standard image acquisition protocols for lower-limb angiography (2 frames/second). The angiograms were acquired in different angulations in the presence of implants to allow better visualization of blood vessels. The image acquisition was identical for DSA and DVA, whereas the post-processing of both these images from the same non-subtracted image series was different.

### Image Processing

DVA and post-processed peak opacification DSA (post-DSA) images were created from the raw radiographic image series using Kinepict Medical Imaging Tool (Kinepict Health Ltd, Hungary) and Syngo software (Siemens Healthcare, Germany), respectively. DVA images were generated retrospectively by calculating the standard deviation for each pixel of the raw image series. Post-DSA images were created on the workstation of Syngo software with available image enhancement tools. Both DSA and DVA images were also motion-corrected using the pixel-shift algorithms of Syngo software and Kinepict Medical Imaging Tool, respectively, and the post-DSA and DVA images were stored as DICOM files and were used for CNR analysis. An experienced radiologist adjusted the contrast and brightness for blinded visual evaluations, and images were saved in a lossless tagged image format (TIF) files for the web-based surveys.

### CNR Analysis

For CNR measurements, regions of interest (ROI) were defined on vessels and background regions using NIH ImageJ [14]. The ROIs were placed in pairs: one vascular ROI and one adjacent background ROI. The angiographic images were categorized into three regions: femoral including the hip joint, popliteal including the knee joint and talocrural below the knee joint. Three pairs of ROIs were placed on every large vascular section. The ROIs placed on the DVA image were readjusted to the corresponding DSA image when there was any geometric difference between the images due to pixel shifting. CNR ratios were calculated for all ROI pairs individually according to the following formula, wherein ${\text{ Mean}}_{{\text{v}}}$ and ${\text{Mean}}_{{\text{b}}}$ referred to mean pixel intensity values of the vascular and background ROI, respectively, and ${\text{Std}}_{{\text{b}}}$ being the background standard deviation.

$${\text{CNR}} = \frac{{\left| {{\text{Mean}}_{{\text{v}}} - {\text{Mean}}_{{\text{b}}} } \right|}}{{{\text{Std}}_{{\text{b}}} }}$$

CNR ratios of corresponding DVA and DSA ROIs were calculated. ROIs were also drawn precisely outlining the implant. Euclidean distance transform (EDT) was used to calculate the mean distances of vascular ROIs to the implant edges. The correlation between CNR ratios and implant distance was also evaluated.

### Qualitative Comparison

Three radiologists with 13, 5 and 4 years of work experience in the field of interventional radiology evaluated the images in a blinded, randomized manner using two online questionnaires, with a 7-day break between the two surveys. Both questionnaires evaluated the aspects of image quality and diagnostic benefit (Tables 1, 2).

**Table 1:**
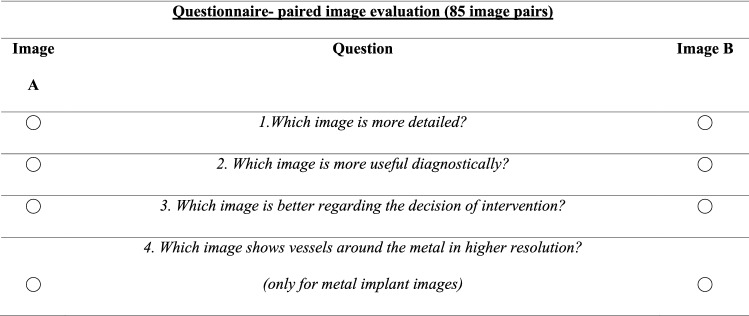
The ‘paired comparison’ questionnaire consisting of four questions for 85 DSA and corresponding DVA image pairs in a random order, without disclosing the type of images to the evaluators

**Table 2:**
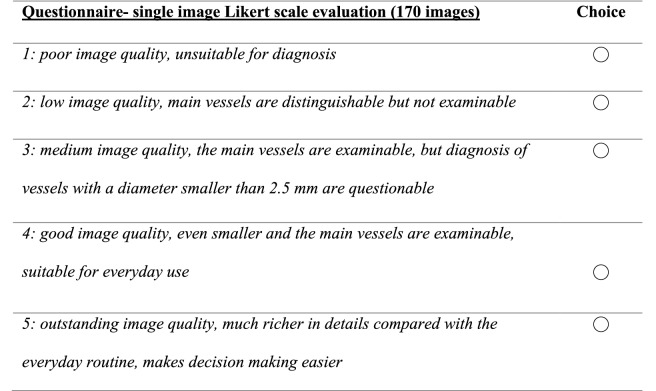
Modified Likert scale for three radiologists: questionnaire for single images (both DSA and DVA in a total of 170 images) in a blinded, randomized manne*r*

### Statistical Analysis

CNR analysis, calculations of the medians and that of confidence intervals were done using Microsoft Excel (Version 16.34, Microsoft, Redmond, WA, USA). Statistical Package for Social Sciences (SPSS—Version 25, IBM Corp., Armonk, NY, USA) was used for statistical analyses; a p value of 0.05 was considered to be significant. Data analysis was done separately for images with and without implants, and an overall analysis for all images was also performed. The paired image evaluation (Table 1) consisted of the quality agreement (percentage of experts in favor of DVA regarding three questions for those without implants and four questions for those with implants) and the interrater agreement. This was first calculated by percent agreement followed by further Fleiss κ test to generate Fleiss κ as well as the corresponding *p *value.

The individual image evaluation (Table 2) consisted of experts’ scores from 1 to 5. The mean and the standard error of mean (SEM) were then calculated. Since the distribution of data was highly asymmetric, the median and the interquartile range were also determined. The Wilcoxon signed-rank test was used for statistical analyses of paired data (DSA vs. corresponding DVA groups) and Mann–Whitney U test for unpaired data (implant vs. no implant groups), respectively.

## Results

### Contrast-to-Noise Ratio Measurements

**Fig. 1:**
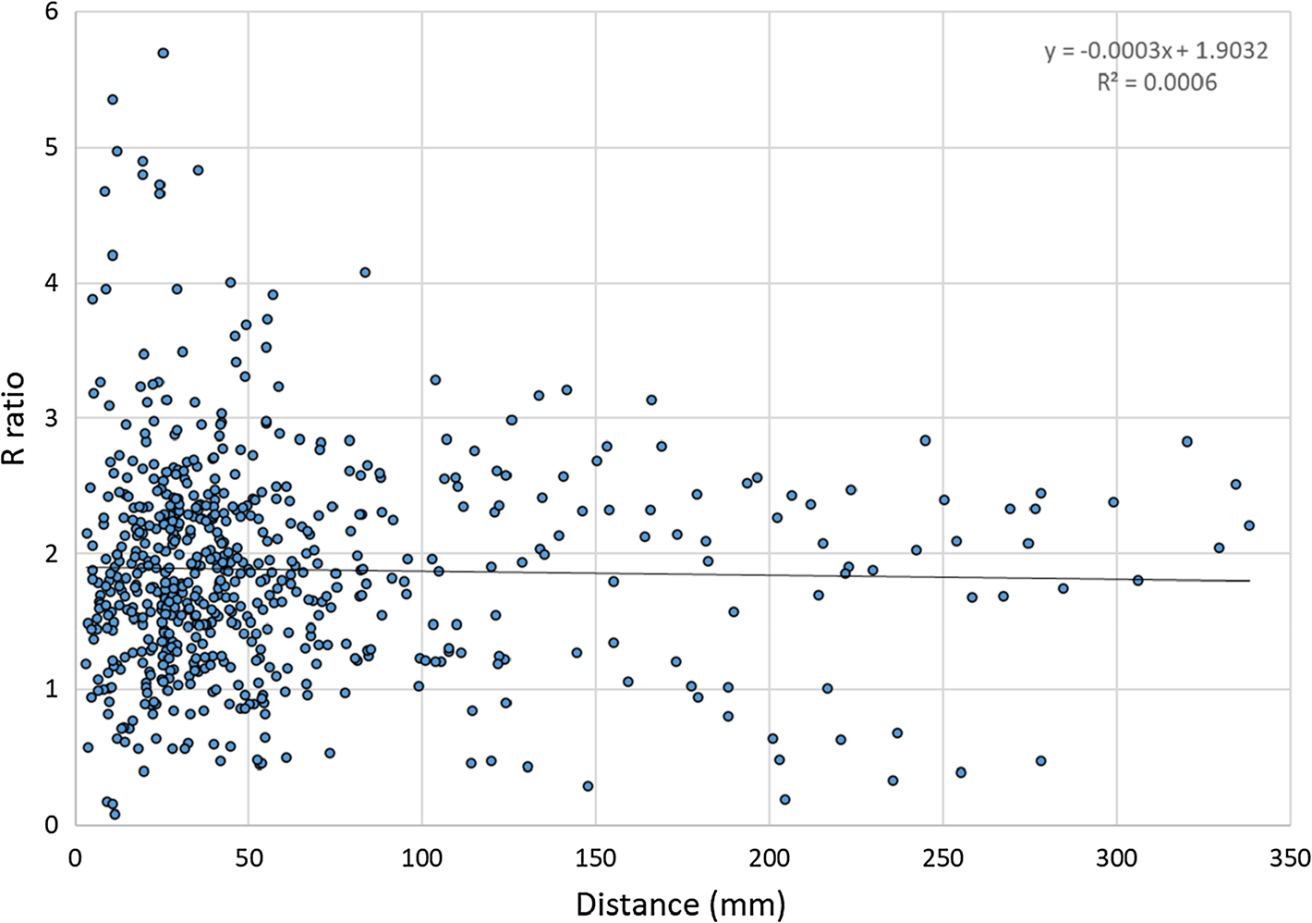
Distance dependence of the *R*-value (CNR_DVA_/CNR_DSA_). Each point represents a ROI pair, x-axis shows the distance of the ROI from the nearest edge of a metal implant, and y-axis shows the *R* values (*R*^2^ in the equation represents the correlation coefficient)

CNR values were calculated and compared in a total of 1252 manually selected ROIs in 37 image pairs containing metal implants, and the R-value of CNR_DVA_/CNR_DSA_ was calculated for every ROI pair. The median ratio of CNR_DVA_/CNR_DSA_ was 1.84 with an interquartile range of 1.35–2.32. The R-value showed no significant correlation with the distance measured from the implant (*p* = 0.548) (Fig. 1).

### Visual Evaluations

#### Paired Comparison

**Fig. 2:**
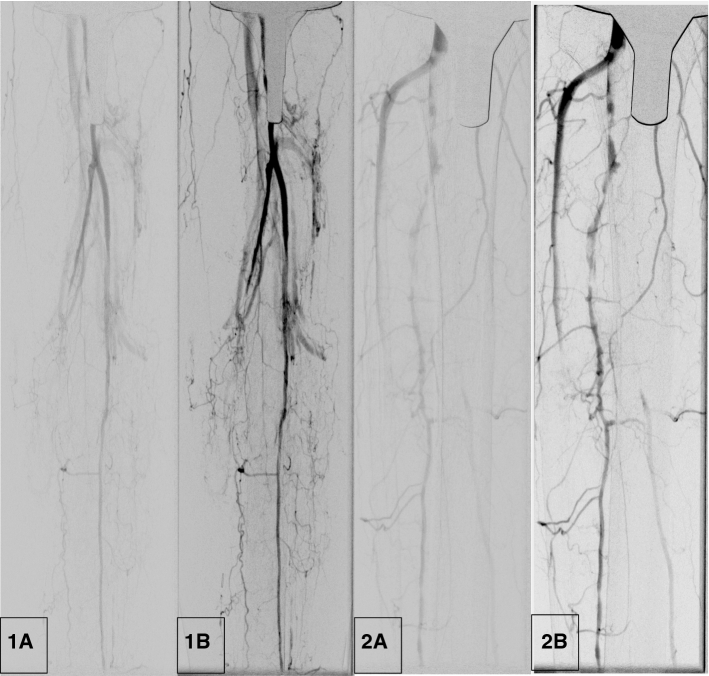
Representative DSA–DVA image pairs. **1A** DSA knee implant, **1B** DVA knee implant, **2A** DSA knee implant, **2B** DVA knee implant

**Fig. 3:**
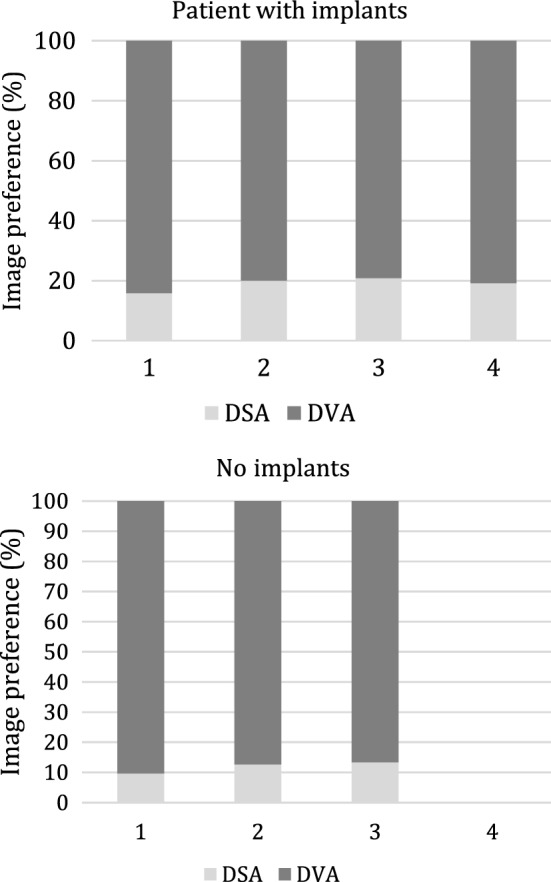
Paired comparison of DVA and DSA images. The bar graph indicates the preference of DSA & DVA images. The numbers below bars represent the different question types (1: details, 2: diagnostic value, 3: ease of therapeutic decisions, 4: blood vessel visibility around the implant). Major gridline unit was changed from 20 to 10 in the upper graph. DSA: digital subtraction angiography; DVA: digital variance angiography

Altogether 85 DSA and corresponding DVA image pairs (Fig. 2) were compared directly using the online questionnaire to evaluate the image quality and the diagnostic benefit. The overall preference of DVA was observed in 84.5% of all images; the interrater agreement for all comparisons was 83.2% with a Fleiss κ value of 0.454 and *p* < 0.001 (Table 3, Fig. 3). With regard to the specific questions (details, diagnostic value, therapeutic decisions), the DVA preference was slightly higher in the implant-free images (quality agreement 86.7–90.4%, interrater agreement 88.1–89.6%, Fleiss κ 0.404–0.487, *p* < 0.001 in all cases*,* Table 3) than in the implant-containing images (quality agreement 79.2–84.2%, interrater agreement 76.7–81.7%, Fleiss κ 0.239–0.408, *p* < 0.005 in all cases). DVA was preferred in 83.75% of comparisons in terms of image quality in the vicinity of implants with a 79.6% interrater agreement and Fleiss κ 0.454 with *p* < 0.001 (Table 3).

**Table 3:** Statistical analysis of the paired image evaluation of DVA & DSA images. Quality agreement represents the preference of DVA images in percentage of the comparisons

Parameter	Number of image pairs	Quality agreement (DVA preference) in %	Percent agreement in %	Fleiss κ	Fleiss κ *p* Value
Question 1 (overall)	85	87.5 (223/255)	85.9	0.357	*p* < 0.001
Implants	40	84.2 (114/120)	81.7	0.312	*P* = 0.001
Ø implants	45	90.4 (122/135)	89.6	0.404	*p* < 0.001
Question 2 (overall)	85	83.9 (214/255)	83.5	0.390	*p* < 0.001
Implants	40	80.0 (96/120)	78.3	0.323	*p* < 0.001
Ø implants	45	87.4 (118/135)	88.1	0.462	*p* < 0.001
Question 3 (overall)	85	83.1 (212/255)	82.7	0.385	*p* < 0.001
Implants	40	79.2 (95/120)	76.7	0.239	*P* = 0.001
Ø implants	45	86.7 (117/135)	88.1	0.487	*p* < 0.001
Question 4 (only implants)	40	80.8 (97/120)	80.8	0.408	*p* < 0.001
Total	85	84.5 (748/885)	83.2	0.454	*p* < 0.001

Percent agreement describes the ratio of concordant decision to all decisions between raters. Interrater agreement was evaluated by percent agreement, corresponding Fleiss κ and *p* value

*DVA *Digital Variance Angiography

### Single-Image Evaluation

**Fig. 4:**
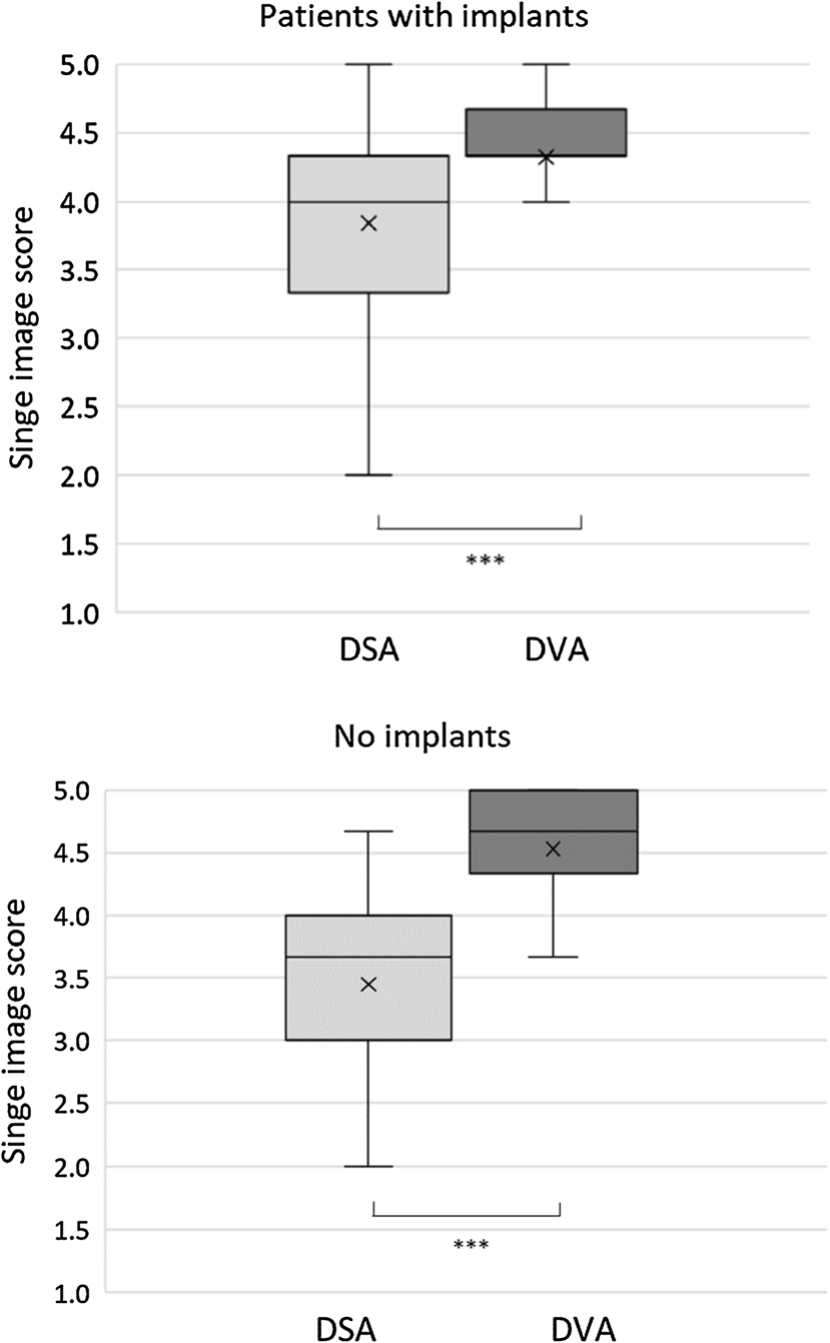
Single-image evaluation of DVA and DSA images and their analysis with Wilcoxon signed-rank test (***p < 0.001). The box and whisker diagrams show the five-grade Likert scale scores, where boxes represent the interquartile range. The line and x within the boxes represent the median and mean value of the groups, respectively. *DSA* Digital Subtraction Angiography; *DVA* Digital Variance Angiography.

Three radiologists evaluated a total of 170 DVA and DSA images in a random blinded manner using the Likert scale (Table 2). The mean, SEM, median and interquartile range are illustrated in Fig. 4. DVA images received significantly higher scores than DSA images in all groups (implant: 4.33 ± 0.09 vs. 3.84 ± 0.12; no implant: 4.53 ± 0.08 vs. 3.45 ± 0.10; all 4.43 ± 0.06 vs. 3.64 ± 0.08; Wilcoxon signed rank *p* < 0.001, Table 4*).* Nevertheless, the difference of image scores was greater in the ‘no-implant’ groups (mean_DVA_-Mean_DSA_ = 1.08) than in the ‘implant’ group (Mean_DVA_-Mean_DSA_ = 0.49), because the DSA images score was significantly lower (Mann–Whitney test: median_DSAøImplant_ = 3.67(*n* = 45); median_DSAimplant_ = 4.0(*n* = 40); *U* = 593.5; *p* < 0.01) and the DVA image score was significantly higher (Mann–Whitney test: median_DVAøImplant_ = 4.67(*n* = 45); median_DVAimplant_ = 4.33(*n* = 40); *U* = 652.5; *p* < 0.05).

**Table 4:** Statistical analysis of single-image evaluation data showing analysis of corresponding DVA–DSA image pairs by Wilcoxon signed-rank test (DVA vs. DSA pairs) and implants vs. no implants by Mann–Whitney U test

Image	*n*	Mean	SEM	Median	Q1–Q3	Wilcoxon	Mann–Whitney
DSA_noimp_	45	3.45	0.10	3.67	3.00–4.00	*p* < 0.001	*P* < 0.01 vs. DSA_Imp_
DVA_noimp_	45	4.53	0.08	4.67	4.33–5.00		*P* < 0.05 vs. DVA_Imp_
DSA_Imp_	40	3.84	0.12	4.00	3.33–4.33	*p* < 0.001	
DVA_Imp_	40	4.33	0.09	4.33	4.33–4.67		
DSA_all_	85	3.64	0.08	3.67	3.00–4.33	*p* < 0.001	
DVA_all_	85	4.43	0.06	4.67	4.33–4.67		

*SEM *standard error of mean, Q1-Q3 interquartile range

## Discussion

DVA is a recently developed technology for image quality improvement in angiographical procedures with great potential of its application in clinical settings with compromised image quality. DVA uses a more elaborate statistical method to extract information about changes than what is done by simple mask subtraction technology of DSA imaging. Measurement of noise and statistical analysis in DVA enables the calculation of standard deviation/variance with functional motion-related information of the X-ray attenuation for every pixel, resulting in the enhancement of contrast agent signal and visualization of vessels [12, 15, 16]. The comparison of DVA to DSA in lower-limb angiography using ICM and CO_2_ has already been reported. Here, DVA is reported to provide a 2.3- to 4.5-fold CNR than DSA, and DVA images proved to be superior in visual evaluations showing 69–85% DVA preference [12, 15].

To the best of our knowledge, angiographic image quality improvement for metal implants with DVA technology has not been reported in the literature. In our study, CNR calculations served as an objective tool and visual quality as a subjective tool to compare the quality of DVA and DSA images in lower-limb angiographies with metal implants. The median CNR_DVA_/CNR_DSA_ ratio (*R*) in our study was almost twofold, reflecting the possibility of acquisition of better image quality even in the direct vicinity of metal implants. This quality reserve in the direct vicinity of metal implants may enable adequate image quality with reduced X-ray dose in the future.

With regard to subjective analyses, DVA images outperformed DSA images both in paired comparison and single-image evaluation surveys. The advantage of DVA images was consistently emphasized more strongly among the implant-free images. This was the consequence of the fact that the DSA score was significantly higher, whereas the DVA score was significantly lower in the implant group. A possible explanation for this finding might be the increased radiation due to automatic beam adjustments with metal implants and a possible activation of additional filters because of the increased scattered radiation [17, 18]. The former might improve the overall image quality of DSA mages, whereas the latter might interfere with the DVA algorithm with slight reduction in its efficacy. Nevertheless, the results clearly show that DVA is superior to DSA even under these conditions and allows a better observation of blood vessels in the direct vicinity of metal parts, as reflected by the 80.8% DVA preference for Question 4 in the paired questionnaire (Table 1). The interrater agreement was moderate at maximum (Fleiss *k* around 0.45), which could be explained by the low number of raters [19]. A higher number of raters should be included in future studies to get a difference or concordance of the interrater agreement.

There are several limitations in our study. The data were obtained in a single center and with a small patient group. One of the experts mentioned a possible disadvantage of DVA. Occasionally, the actual pathologies, for example atherosclerotic plaques or the degree of stenosis, could be masked with the overall increase in contrast and visualization of the whole vessel. Our study involved a retrospective analysis of previously collected images, which were diagnostically useful. With the installation of DVA technology in the operating room, real-time DVA data are provided along with the DSA images, as reported by Gyánó *et al.* [20]. The two image types could then be used in a complementary manner and thereby support the radiologist in the decision-making process, instead of using each of these technologies alone. Despite these limitations, the results of this pilot study clearly demonstrate the potential of DVA technology in lower-limb angiographies with metal implants for better image quality and reduction in radiation dose. Further prospective studies with a larger patient group in a multicentric setup could validate these results in the future.

## Conclusions

The objective CNR measurement and subjective visual evaluation data demonstrate that DVA provides significant improved image quality in lower-limb angiography with metal implants, compared to DSA imaging. The observed quality reserve suggests that this approach could reduce radiation exposure for angiography with metal implants without compromising the diagnostic value and image quality of angiograms. Further clinical studies could validate these results in the future.
